# Comparison of refractive and visual acuity results after Contoura^®^ Vision topography-guided LASIK planned with the Phorcides Analytic Engine to results after wavefront-optimized LASIK in eyes with oblique astigmatism

**DOI:** 10.1371/journal.pone.0279357

**Published:** 2022-12-19

**Authors:** Phillip Brunson, Paul M. Mann, Paul Michael Mann, Richard Potvin

**Affiliations:** 1 Mann Eye Institute and Laser Centers, Houston, TX, United States of America; 2 Science in Vision, Bend, OR, United States of America; University of Toronto, CANADA

## Abstract

**Purpose:**

To compare visual acuity and refractive results between topography-guided laser in situ keratomileusis (LASIK) planned with the Phorcides Analytic Engine (PAE) to results after wavefront-optimized (WFO) LASIK in subjects with preoperative oblique astigmatism in their manifest refraction.

**Methods:**

This was a retrospective chart review of clinical results from eyes treated with topography-guided LASIK planned with PAE compared to eyes treated with WFO LASIK using the same Wavelight^®^ excimer laser system. All included subjects had preoperative oblique astigmatism. Residual refractive error and visual acuity (uncorrected and corrected) were the measures of interest, at the visit closest to 90 days postoperative.

**Results:**

A matched data set from 100 WFO and 97 PAE eyes was extracted from clinical records. At the postoperative visit the PAE group showed lower residual refractive cylinder (p = 0.04), uncorrected distance visual acuity (UDVA) (-0.06 PAE vs. -0.02 WFO, p < 0.01) and distance corrected visual acuity (CDVA) (p < 0.01). The percentage of eyes with a mean refraction spherical equivalent (MRSE) magnitude within 0.25 D and 0.50 D of plano was statistically significantly higher in the PAE group (p = 0.04 and 0.01, respectively). A statistically significantly higher percentage of eyes in the PAE group had UDVA better than or equal to -0.10 logMAR (20/16 Snellen, 36% vs 22%, p = 0.04). More eyes gained CDVA after surgery in the PAE group (53% vs 32%, p < 0.01). There were five enhancements in the WFO group versus none in the PAE group, a statistically significant difference (p = 0.03).

**Conclusions:**

Visual acuity and refractive outcomes after LASIK using PAE in eyes with oblique astigmatism in their preoperative refraction were statistically significantly better than those obtained when WFO treatment was used. The number of refractive outliers and the number of retreatments were also significantly lower with PAE treatment.

## Introduction

Laser in situ keratomileusis (LASIK) is one of the most common corneal surgical refractive procedures in use today, providing excellent results with a good safety profile [1]. However, a small percentage of patients may be dissatisfied. One of the recognized contributors to dissatisfaction after LASIK is residual refractive error, particularly residual astigmatism, or irregular astigmatism after surgery [2, 3]. In these instances, a retreatment is often helpful in improving the patient’s level of satisfaction [3]. Treating astigmatism can be challenging because while the manifest astigmatism is generally the target treatment, results appear slightly less predictable if the source of astigmatism is not the anterior cornea [4]. The orientation of the astigmatism may also affect the predictability of results.

Astigmatism may be classified by its axis as with-the-rule (WTR), against-the-rule (ATR), or oblique. Generally, WTR is more common than ATR and ATR is more common than oblique astigmatism. Oblique astigmatism is estimated to account for 10–20% of all astigmatism (corneal and manifest) in the general population across all age groups [5, 6]. Compared to ATR and WTR, oblique astigmatism appears to cause more difficulties in binocular perception [7], may decrease reading performance [8], result in lower distance visual acuity [8], and reduced contrast (compared to WTR) [9]. In addition, oblique astigmatism is more likely to be irregular than WTR or ATR astigmatism [10]. Subjective blur limits for oblique astigmatism have also been reported to be lower (worse) than when astigmatism is WTR [11].

The orientation of astigmatism is also known to be a factor influencing refractive surgery results. Outcomes after cataract surgery and toric IOL implantation tend to be more variable in eyes with oblique astigmatism relative to eyes with ATR or WTR astigmatism [12]. One study of correcting astigmatism with a corneal refractive procedure suggests results are also more variable when the astigmatism is oblique [13]. We could locate no studies of LASIK outcomes where results were stratified by astigmatism orientation.

Topography-guided LASIK procedures were originally developed as a method to correct irregular astigmatism [14]. Given that oblique astigmatism tends to be more irregular [10], the ability of topography-guided LASIK procedures to correct oblique astigmatism may be better than for other LASIK procedures, such as wavefront-optimized (WFO) LASIK. The Contoura^®^ Vision procedure (Contoura, Alcon Vision LLC, Fort Worth, TX, USA) is a topography-guided LASIK procedure that is used to treat cases of both irregular and regular astigmatism. Clinical trial results submitted for the Food and Drug Administration (FDA) approval of Contoura reported good visual outcomes 12 months postoperatively [15]. This clinical trial included a restriction in the degree to which topographic and refractive astigmatism could differ, and treatment was based on the manifest refraction [15].

When the manifest and topographic astigmatic magnitudes and axes differ significantly, the treatment may be based on the manifest refractive cylinder, the topographic astigmatism or some combination of the two; the optimal treatment based on these two measures remains a subject of debate. Studies have demonstrated good results with planning based on the manifest cylinder [16]. Results using the topographic astigmatism and axis alone appeared to be less satisfactory [17]. More recently, results using the Phorcides Analytic Engine (PAE, Phorcides LLC, North Oaks, MN, USA) have also been shown to be effective [18–20]. PAE uses software originally developed for geographic imaging to characterize the shape of the anterior cornea. Proprietary algorithms then generate an objective treatment recommendation for sphere and cylinder based on this, as well as the anterior and posterior corneal astigmatism, and the patient’s refractive data.

This retrospective study compares clinical outcomes between a) WFO treatments and b) treatments using Contoura with surgical planning based on PAE (with the same laser) in eyes with preoperative oblique astigmatism.

## Material and methods

This study was a double arm, retrospective chart review of clinical outcomes from two surgeons at one site. The study was approved by an institutional review board (Salus IRB, Austin, TX, USA), which also granted a waiver of informed consent. The study was conducted in compliance with Good Clinical Practice (GCP), the tenets of the Declaration of Helsinki, and International Harmonization (ICH) guidelines. The study was non-interventional, so registration with a clinical trial registry was not required.

Eligible charts were from patients that received bilateral on-label treatment of refractive error with either the Contoura topography-guided treatment profile planned using PAE, or a WFO ablation profile. Subjects had to have clinical results from 1–6 months postoperatively available and had to have a preoperative manifest refraction with any amount of oblique astigmatism (31 to 59 degrees or 121 to 149 degrees). If more than one exam was available, the exam closest to 3 months was used. Patients with clinically significant ocular pathology (other than residual refractive error), previous refractive surgery, hyperopia, abnormal topographies (e.g., keratoconus), a calculated residual stromal bed thickness less than 250 μm, or other ocular pathology that might affect the results of LASIK were excluded. Patients with suboptimal surgical outcomes not related to the method of treatment (eg flap dislocation) were excluded.

The target sample size was 100 subjects in each group. For the WFO group, clinical records were reviewed from April 2018 backward to identify suitable subjects/eyes that met the inclusion and exclusion criteria above. Similarly, PAE records were reviewed from June 2019 (when PAE was widely adopted in the clinic) forward. Eligible records were de-identified and the relevant chart information was extracted. Preoperative data included sex, age, refractive error, corrected visual acuity, and surgical planning and treatment. Postoperative data included refractive error and corrected/uncorrected visual acuity. All visual acuity data were recorded in Snellen notation; results were converted to logMAR for analysis.

For the PAE surgeries, corneal topographies were obtained using the WaveLight^®^ Topolyzer Vario^™^ (Alcon Vision, LLC, Fort Worth, TX, USA) and transferred to the Contoura software. All LASIK procedures were performed by two experienced surgeons (PM, PM II). Corneal flaps were created using the WaveLight^®^ FS200 femtosecond laser (Alcon Vision, LLC, Fort Worth, TX, USA) set for a 9.0 mm diameter flap with a 110 μm thickness. The corneal ablation was completed using the WaveLight^®^ EX500 excimer laser (Alcon Vision, LLC, Fort Worth, TX, USA) set for a 6.50 mm optical zone.

The statistical software program Statistica (version 12, TIBCO Software Inc., Palo Alto, CA, USA) was used to perform all statistical analyses. Parametric variables were compared using an analysis of variance (ANOVA), while non-parametric variables were compared using a Chi-squared test. All analyses used a Type I error rate of p < 0.05.

## Results

A total of 100 eyes were successfully extracted from the clinical record and verified for inclusion in the WFO group. Only 97 qualified eyes in the PAE group were identified, due to a lower total number available. Table 1 summarizes the demographics and preoperative refractive status for the two groups. As can be seen, the groups were well matched for age and preoperative refractive status. The PAE group had a slightly shorter follow up. No adverse events or serious adverse events were reported to the principal investigator during the course of this data extraction.

**Table 1:** Summary demographics and preoperative data.

	WFO	PAE	p-value
Patients/eyes	85/100	83/97	
Age (years)	34 ± 9 (22 to 59)	34 ± 8 (20 to 58)	0.85
MRSE (D)	-3.67 ± 1.90 (-9.00 to -0.50)	-4.10 ± 1.67 (-7.63 to -0.38)	0.09
Refractive cylinder (D)	-0.78 ± 0.55 (-3.00 to -0.25)	-0.69 ± 0.45 (-2.25 to -0.00)	0.20
CDVA (logMAR)	-0.01 ± 0.03 (-0.10 to 0.04)	-0.01 ± 0.04 (-0.14 to 0.10)	0.59
Follow-up duration (days)	71 ± 33 (28 to 154)	61 ± 30 (28 to 138)	0.02

Abbreviations: MRSE—mean refraction spherical equivalent; D—diopter; logMAR—log of the minimum angle of resolution; CDVA—corrected distance visual acuity

Table 2 summarizes the postoperative refractive and visual acuity data for the two groups. For all but the mean refraction spherical equivalent (MRSE) the differences between the groups were statistically significant. However, as can be seen, the mean refractive cylinder difference is clinically insignificant. Mean uncorrected visual acuity (UDVA) was two letters better in the PAE group (just under half a line of logMAR acuity).

**Table 2:** Summary postoperative refractive and visual acuity data.

	WFO	PAE	p-value
MRSE (D)	-0.02 ± 0.27 (-1.00 to 0.75)	0.04 ± 0.18 (-0.50 to 0.50)	0.06
Refractive cylinder (D)	-0.13 ± 0.31 (-1.75 to -0.00)	-0.06 ± 0.15 (-0.75 to 0.00)	0.04
UDVA (logMAR)	-0.02 ± 0.07 (-0.12 to 0.16)	-0.06 ± 0.06 (-0.20 to 0.10)	< 0.01
CDVA (logMAR)	-0.03 ± 0.05 (-0.12 to 0.10)	-0.06 ± 0.06 (-0.20 to 0.06)	< 0.01

Abbreviations: MRSE—mean refraction spherical equivalent; D—diopter; logMAR—log of the minimum angle of resolution; UDVA—uncorrected distance visual acuity; CDVA—corrected distance visual acuity

**Fig 1:**
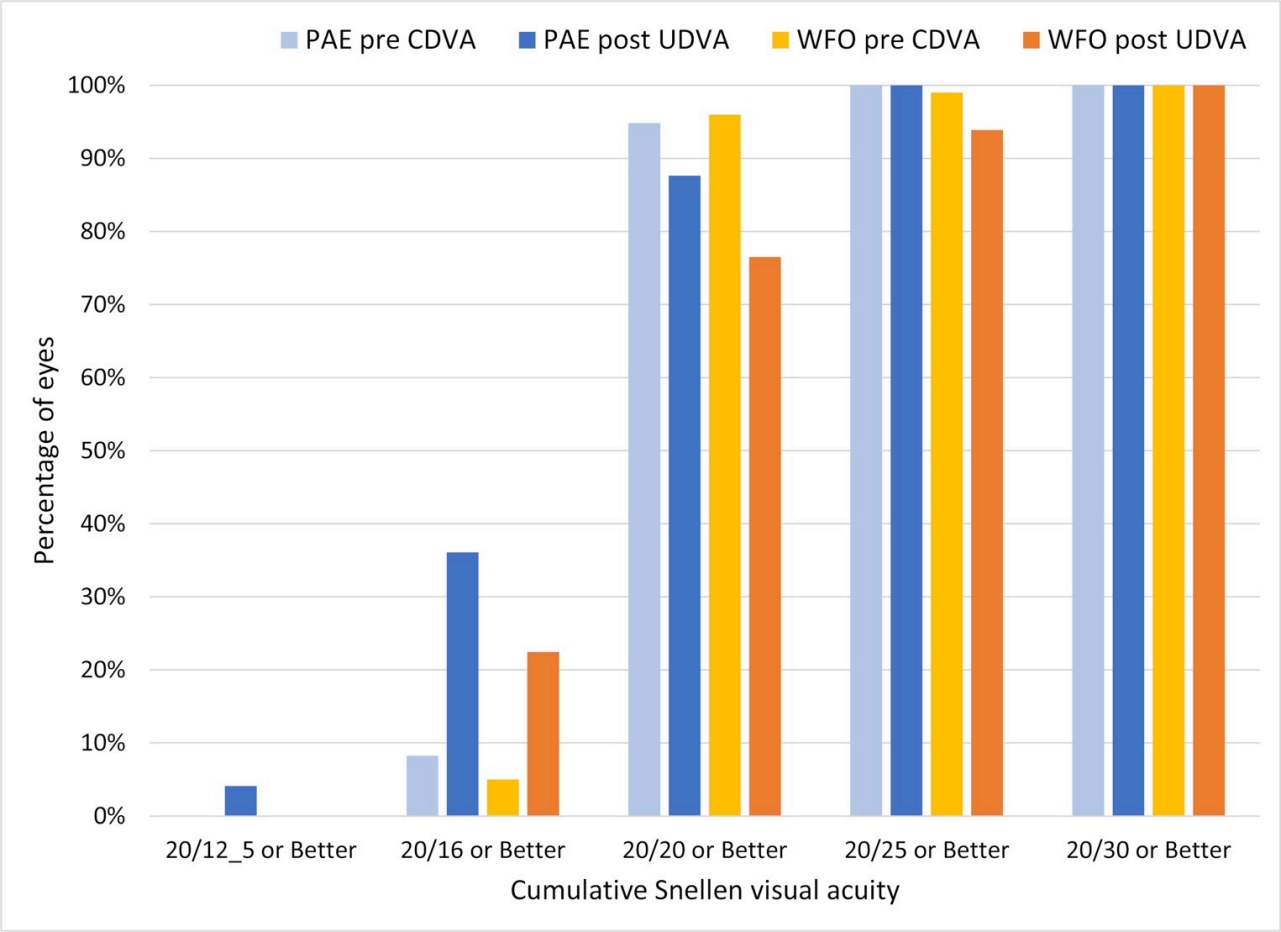
Preoperative corrected distance visual acuity (CDVA) and postoperative uncorrected distance visual acuity (UDVA) by treatment. Abbreviations: WFO–wavefront optimized, PAE–Phorcides Analytic Engine.

Fig 1 is a standard refractive surgery outcomes graph that shows the relationship between preoperative corrected distance visual acuity (CDVA) and postoperative UDVA. As can be seen, in both groups there were more eyes with 20/16 UDVA postoperative than 20/16 CDVA preoperative. The percentage of eyes with 20/16 UDVA postoperative was higher for the PAE group (36% vs. 22%, p = 0.04).

**Fig 2:**
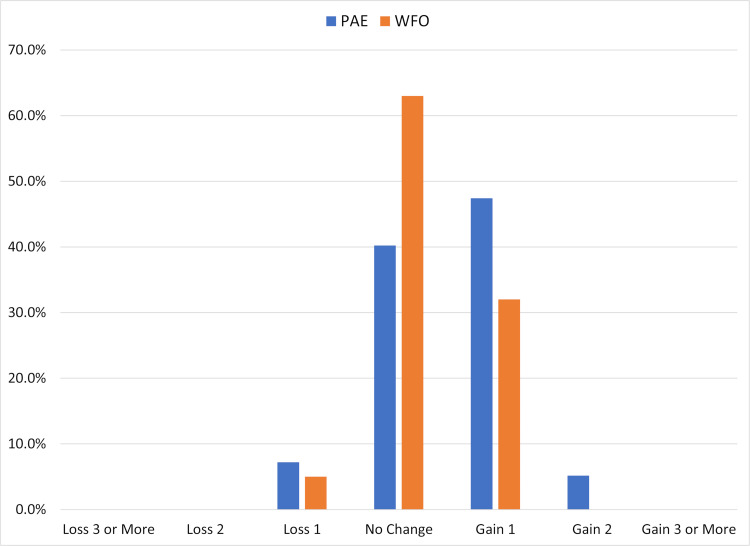
Preoperative to postoperative change in corrected distance visual acuity (CDVA). Abbreviations: WFO–wavefront optimized, PAE–Phorcides Analytic Engine.

Fig 2 is another standard refractive surgery outcomes graph that shows the change in CDVA from preoperative to postoperative. No eyes in either group lost more than one line of CDVA. The percentage of eyes gaining a line or more of CDVA was significantly higher in the PAE group relative to the WFO group (53% vs 32%, p < 0.01). There was no difference between groups in the percentage of eyes that lost one line of CDVA (7% vs 5%, p = 0.52).

Table 3 shows a categorization of the refractive data from both groups. The percentage of eyes with an MRSE magnitude within 0.25 D and 0.50 D of plano was statistically significantly higher in the PAE group. There were no statistically significant differences in the percentage of eyes with ≤ 0.25 D or ≤ 0.50 D of refractive cylinder. Looking at a combined effect, about 10% more eyes in the PAE group had a refraction within 0.50 D of plano with ≤ 0.50 D of refractive cylinder; this result was statistically significant. A statistically significantly higher percentage of eyes in the PAE group also had UDVA better than or equal to -0.10 logMAR (20/16) and 0.00 logMAR (20/20) relative to the WFO group. Note that with more than 85% of eyes in both groups having postoperative refractive cylinder ≤ 0.25D, a polar plot of results was not considered helpful to the analysis.

**Table 3:** Categorized postoperative refractive and visual acuity results.

		WFO	PAE	p-value
MRSE magnitude (D)	≤ 0.25	78 (78%)	88 (91%)	0.04
	≤ 0.50	94 (94%)	97 (100%)	0.01
Refractive cylinder magnitude (D)	≤ 0.25	86 (86%)	90 (93%)	0.12
	≤ 0.50	94 (94%)	96 (99%)	0.06
MRSE magnitude ≤ 0.50 D with ≤ 0.50 D of refractive cylinder		90 (90%)	96 (99%)	0.01
UDVA ≤ -0.1 logMAR (20/16)		22 (22%)	35 (36%)	0.04
UDVA ≤ 0.0 logMAR (20/20)		75 (75%)	85 (88%)	0.04

Abbreviations: MRSE—mean refraction spherical equivalent; D—diopter; logMAR—log of the minimum angle of resolution; UDVA—uncorrected distance visual acuity

There were five enhancements in the WFO group, two of which were related to postoperative astigmatism of 1.0 D or more. There were no enhancements in the PAE group. This difference was statistically significant (Fisher’s Exact test, p = 0.03). All the enhancements were performed after the visits from which the study data were extracted.

## Discussion

This study attempted to evaluate the outcomes of PAE versus WFO-assisted LASIK surgeries in eyes with preoperative oblique astigmatism in the manifest refraction. The hypothesis was that a topography-guided treatment might have some advantages in this population, as oblique astigmatism is often associated with irregular astigmatism [10].

There have been several studies comparing the outcomes of topography-guided LASIK to those of WFO LASIK. In a contralateral eye study using the LYRA protocol (Layer Yolked Reduction of Astigmatism), Ozulken et al found no significant difference in visual acuity and refractive status postoperatively between the topography guided and WFO eyes [21]. They reported more residual astigmatism in both groups than was found in the current study, and a lower percentage of eyes with 20/16 or better visual acuity. Tiwari et al reported similar outcomes in their study, though the method of calculating the topography-guided treatment was not specified [22]. Another topography guided contralateral eye study by Shetty et al also showed no differences between topography-guided and WFO refractive and acuity results, though their topography guided results were not as good as the results here in terms of either refractive cylinder or UDVA [23]. As with Tiwari, the calculation method for determining the topography guided treatment was not specified. Zhang et al reported results based on the TMR (Topography-modified refraction) approach designed by Kanellopoulos and found 8% of subjects had 0.50 D or more of refractive astigmatism 6 months postoperatively [24]. Similarly, Kim et al found higher residual astigmatism with the TMR method, but no significant differences between topography guided LASIK based on the manifest refraction, the TMR method or standard WFO treatment [25]. Visual acuity and refractive outcomes for either topography guided surgery group were not as good as seen in the current study. Finally, Cheng et al conducted a meta-analysis and reported that topography guided LASIK appeared to improve refractive accuracy relative to WFO LASIK [26]. It is worth noting that in all the above studies the results with WFO and topography guided LASIK were very good, making it difficult to detect incremental improvements in performance. In addition, none of the results above included PAE as a treatment planning method. Studies with larger sample sizes are advocated.

There have been prior studies of PAE as well, though none compared PAE results to WFO results [18, 19]. PAE results here appear consistent with those reported in these previous studies. It must be noted that comparisons of data across studies is always confounded by such considerations as test conditions (e.g., lighting) and variations in test procedures.

This study has several limitations. It was a retrospective chart review. Extracting information from the clinical record meant that only standard outcomes data (i.e., refraction and visual acuity) were available. Acuity was measured in Snellen notation, which necessitated conversion to logMAR for analytical purposes; an ETDRS or similar logMAR chart for testing is the preferred method of collecting VA data in studies. A slightly shorter follow-up time in the PAE group was noted, but the difference was deemed clinically unimportant–all included eyes had data from 4 weeks to 6 months postoperative. If there was any bias it would have likely been in the direction of favoring the WFO group [27]. Finally, results are limited to those obtained by two surgeons at one site.

In summary, refractive and visual acuity outcomes after topography guided LASIK using PAE in eyes that had oblique astigmatism in their preoperative refraction were statistically significantly better than those obtained when WFO treatment was used, though the differences were modest. Most notable was that topography guided LASIK using PAE showed a statistically higher percentage of patients achieving 20/20 and 20/16 vision than was achieved with WFO treatment, and a higher percentage of patients in the PAE group gained CDVA after surgery. The number of refractive outliers and the number of retreatments were also significantly lower when PAE was used. These results suggest that topography guided LASIK using PAE may be preferred over WFO for eyes with preoperative oblique astigmatism.

## Supporting information

S1 File This is the anonymized raw data file, in excel format. (XLSX)
